# Some Remarks on the Boundary of Thermodynamic Stability

**DOI:** 10.3390/e25070969

**Published:** 2023-06-23

**Authors:** Alexander Toikka, Georgii Misikov, Maria Toikka

**Affiliations:** Department of Chemical Thermodynamics & Kinetics, Saint Petersburg State University, Universitetsky pr. 26, Peterhof, Saint Petersburg 198504, Russia; st062450@student.spbu.ru (G.M.); m.toikka@spbu.ru (M.T.)

**Keywords:** stability conditions, boundary of stability, neutral equilibrium, Gibbs–Duhem equation

## Abstract

In this paper, we have considered some elements of the classical phenomenological theory of thermodynamic stability, which seem controversial and ambiguous. The main focus is on the conditions of the stability boundary; a new version of the derivation of the relations defining the specified boundary is proposed. Although the final results, in general, coincide with the classical relations, the described approach, from our point of view, provides a clearer and more accurate idea of the stability conditions and their boundaries.

## 1. Introduction

Thermodynamic stability conditions are a well-developed field of research, which has been equally studied in physicochemical and mathematical terms. The inclusion of the concept of stability in the apparatus of general thermodynamics is largely due to the work of Gibbs, whose merit is the very definition of thermodynamic equilibrium and its stability [1]. The initial basis of the thermodynamic theory formally coincides with the concepts of equilibrium and stability in analytical mechanics and, more precisely, with the extreme principles of mechanics. The main difference between the thermodynamic stability analyses is related to the involvement of the consequences of the second law of thermodynamics, both for the formulation of the stability criteria themselves and for particular conclusions, and important physicochemical results. One of the problems from our point of view, which raises some questions, is the analysis of the boundaries of thermodynamic stability. In this paper, alternative conditions for defining this boundary are considered.

## 2. Results and Discussion

An obvious condition of the stability boundary is that the expression, which, according to Gibbs, is a stability criterion, is equal to zero [1]. Next, we consider the stability condition with respect to the so-called continuous state changes, that is, infinitesimal changes. Such a restriction, as indicated, for example, in [2], allows us to obtain more general conclusions since it includes a study of the stability of both stable and metastable states. Otherwise, the analysis of metastable states, by their definition, requires the involvement of data on the limits within which these states exist. In addition, it is sufficient to consider the stability of homogeneous states; their analysis easily extends to heterogeneous systems, with some additional nuances [2,3]. In a fairly general form, this stability condition, following Gibbs [1], can be represented as

(1) $$U^{\prime \prime }-\underset{i}{\sum }X_{i}^{\prime }Y_{i}^{\prime \prime }>0,$$

where U is the internal energy; $X_{i}$ is the intensive parameter; $Y_{i}$ is the conjugate extensive parameter; values with one stroke refer to the state being tested for stability values, with two strokes refer to the neighboring virtual state. We emphasize that, unlike a number of studies, for example [1,2,3], in which it is further assumed that the neighboring state is a real infinitely close equilibrium state, we do not introduce such restrictions on the virtual state. In other words, the parameters of an infinitely close virtual state do not necessarily correspond to the parameters of any of the real equilibrium neighboring states and may belong to a non-equilibrium state. Apparently, this is more in line with Gibbs’ original idea of the stability criterion [1]. Note also that in Equation (1), as in other stability studies, including [1], the validity of the integral forms of fundamental equations is accepted, which is a certain known limitation. The case of equilibrium between the initial and virtual phases, that is, the corresponding phase equilibrium, is also excluded. For simplicity and clarity, we further limit ourselves to the “conventional set” of thermodynamic parameters and present (1) in equivalent forms [1]:

(2) $$U^{\prime \prime }-U^{\prime }>T^{\prime }(S^{\prime \prime }-S^{\prime })-P^{\prime }\left(V^{\prime \prime }-V^{\prime }\right)+\mu_{1}^{\prime }\left(m_{1}^{\prime \prime }-m_{1}^{\prime }\right)+\cdots +\mu_{n}^{\prime }\left(m_{n}^{\prime \prime }-m_{n}^{\prime }\right)$$

or

(3) $$-T^{\prime }S^{\prime \prime }+P^{\prime }V^{\prime \prime }-\mu_{1}^{\prime }m_{1}^{\prime \prime }-\cdots -\mu_{n}^{\prime }m_{n}^{\prime \prime }+T^{\prime \prime }S^{\prime \prime }-P^{\prime \prime }V^{\prime \prime }+\mu_{1}^{\prime \prime }m_{1}^{\prime \prime }+\cdots +\mu_{n}^{\prime \prime }m_{n}^{\prime \prime }>0$$

where T is absolute temperature, S —entropy, V —volume, P —pressure, $m_{i}$ is the amount of substance i , and $\mu_{i}$ —the chemical potential of this substance. Note that the pressure in Equation (1) should be represented as (−P) , that is, with a minus sign.

Equation (3) could be transformed into a known form [3]

(4) $$\left(T^{\prime \prime }-T^{\prime }\right)S^{\prime \prime }-\left(P^{\prime \prime }-P^{\prime }\right)V^{\prime \prime }+\left(\mu_{1}^{\prime \prime }-\mu_{1}^{\prime }\right)m_{1}^{\prime \prime }+\cdots +\left(\mu_{n}^{\prime \prime }-\mu_{n}^{\prime }\right)m_{n}^{\prime \prime }>0$$

that actually coincides with one of the Gibbs stability conditions ([1], p. 107, formula (148)). Since the differences in parentheses in (4) are infinitesimal exact differences, neglecting infinitesimal higher orders, we turn to differentials and write

(5) $$S^{\prime \prime }dT-V^{\prime \prime }dP+m_{1}^{\prime \prime }d\mu_{1}+\cdots +m_{n}^{\prime \prime }d\mu_{n}\geq 0$$

The sign ≥ in Equation (5) appeared due to the fact that, unlike Equation (4), the contribution of infinitesimal higher orders is not taken into account in Equation (5). As a result, (5) is only a necessary stability condition of the phase (‘) with respect to continuous state changes. 

It is obvious that the left side of (5) coincides in form with—the left side of the Gibbs–Duhem equation [2,4,5]:

(6) $$SdT-VdP+m_{1}d\mu_{1}+\cdots +m_{n}d\mu_{n}=0.$$

Nevertheless, the difference between (5) and (6) is quite obvious. Equation (5), as well as Equation (4), characterizes a virtual perturbation of a state, which in general is not equilibrium. Formula (6) refers to the equilibrium change of state. Accordingly, the stability analysis cannot be carried out on the basis of condition (6). In the literature, this important point is ignored, which leads to significant errors. For example, in a book ([4], chapter 15, paragraph 14), an erroneous derivation is given, and the conclusion about zeroing the stability determinant (at T, P=const) : partial derivatives in the Jacobian are calculated from the Gibbs–Duhem equation, which, as indicated above, is unacceptable. We also note beforehand that Equation (6) is quite compatible with one of the conditions of the stability boundary considered by Gibbs, namely, the relation determining the neutral equilibrium [1,3]:

(7) $$dT=dP=d\mu_{1}=\cdots d\mu_{n}=0.$$

Another important aspect of the analysis of the stability of a thermodynamic system is associated with the introduction or selection of a parameter that allows excluding changes associated only with an increase/decrease in the mass of the system, and not a change in its state, that is, intensive parameters. This “scale factor” [6] may be, for example, the volume or total mass of the system. Such a selection of one of the parameters (in the case of volume) in the stability analysis based on Equations (3)–(5) is obviously superfluous since all extensive parameters in these relationships are fixed. Nevertheless, in all works, for example [1,2,3,7,8,9], when considering stability, the specified “scale factor” is introduced in different forms, possibly in order to circumvent the condition of “zeroing” the determinant. It is also not entirely clear what Gibbs meant when discussing the stability condition

(8) $$\Delta U>T\Delta S-P\Delta V+\mu_{1}\Delta m_{1}+\cdots +\mu_{n}\Delta m_{n}$$

([1], p. 106, Equation (145)), and indicated that “If only the quantity of the body which determines the value of the variables should vary and not its phase, the value of the first member of (145) would evidently be zero”. Obviously, the value of the left side (8), that is, the change in the internal energy of the system, when its mass changes, will not be zero, but will also change. Here, as before, the formulas from Gibbs’ works are given in accordance with modern notation.

We have already noted above that the determinant that defines stability, in accordance with the condition $\delta^{2}U>0$ , that is, the Hessian (for the set of n+2 pairs of parameters chosen above)

(9) $$\left|\begin{array}{cccc} \frac{\partial^{2}U}{\partial Y_{1}^{2}} & \frac{\partial^{2}U}{\partial Y_{1}\partial Y_{2}} & \cdots & \frac{\partial^{2}U}{\partial Y_{1}\partial Y_{n+2}}\\ \frac{\partial^{2}U}{\partial Y_{2}\partial Y_{1}} & \frac{\partial^{2}U}{\partial Y_{2}^{2}} & \cdots & \frac{\partial^{2}U}{\partial Y_{2}\partial Y_{n+2}}\\ \vdots & \vdots & \ddots & \vdots \\ \frac{\partial^{2}U}{\partial Y_{n+2}\partial Y_{1}} & \frac{\partial^{2}U}{\partial Y_{n+2}\partial Y_{2}} & \cdots & \frac{\partial^{2}U}{\partial Y_{n+2}^{2}} \end{array}\right|>0$$

is generally not zero, since the derivatives in (9) are taken when the equilibrium is perturbed and, accordingly, are not limited by the relationships

(10) $$\begin{array}{c} Y_{1}\frac{\partial^{2}U}{\partial {Y_{1}}^{2}}+Y_{2}\frac{\partial^{2}U}{\partial Y_{1}\partial Y_{2}}+\cdots +Y_{n+2}\frac{\partial^{2}U}{\partial Y_{1}\partial Y_{n+2}}=0\\ Y_{1}\frac{\partial^{2}U}{\partial Y_{2}\partial Y_{1}}+Y_{2}\frac{\partial^{2}U}{\partial Y_{2}^{2}}+\cdots +Y_{n+2}\frac{\partial^{2}U}{\partial Y_{2}\partial Y_{n+2}}=0\\ \vdots \\ Y_{1}\frac{\partial^{2}U}{\partial Y_{n+2}\partial Y_{1}}+Y_{2}\frac{\partial^{2}U}{\partial Y_{n+2}\partial Y_{2}}+\cdots +Y_{n+2}\frac{\partial^{2}U}{\partial Y_{n+2}^{2}}=0 \end{array}\},$$

that are consequences of the Gibbs–Duhem equation

(11) $$Y_{1}d\frac{\partial U}{\partial Y_{1}}\;\;+Y_{2}d\frac{\partial U}{\partial Y_{2}}+\cdots +Y_{n+2}d\frac{\partial U}{\partial Y_{n+2}}=0.$$

It is the particular variant of the relationships in (10) that were used in [4] to prove, for special cases ( T, P=const) , the identical equality to zero of the determinant of the quadratic form of the increments of extensive parameters. Respectively, the proof given in [4] is incorrect.

Accordingly, since it is the determinant in (9) that vanishes first, and not its minors, the stability boundary is equal to zero. It is important to note that in the above conclusions, the condition of constancy of one of the parameters or their combination, for example, the total amount of substances, was not used. The correctness of the result is also obvious from the fact that (9) includes derivatives of intensive quantities (by extensive parameters).

Finally, we consider another condition of the stability boundary obtained by converting the determinant in (9) into a product of partial derivatives [1,10,11]

(12) $$\left|\begin{array}{cccc} \frac{\partial^{2}U}{\partial Y_{1}^{2}} & \frac{\partial^{2}U}{\partial Y_{1}\partial Y_{2}} & \cdots & \frac{\partial^{2}U}{\partial Y_{1}\partial Y_{n+2}}\\ \frac{\partial^{2}U}{\partial Y_{2}\partial Y_{1}} & \frac{\partial^{2}U}{\partial Y_{2}^{2}} & \cdots & \frac{\partial^{2}U}{\partial Y_{2}\partial Y_{n+2}}\\ \vdots & \vdots & \ddots & \vdots \\ \frac{\partial^{2}U}{\partial Y_{n+2}\partial Y_{1}} & \frac{\partial^{2}U}{\partial Y_{n+2}\partial Y_{2}} & \cdots & \frac{\partial^{2}U}{\partial Y_{n+2}^{2}} \end{array}\right|=\prod_{i=1}^{n+2}{\left(\frac{\partial^{2}U}{\partial Y_{i}^{2}}\right)}_{\;\frac{\partial U}{\partial Y_{j=1,\ldots i-1}},{\;Y}_{k=i+1,\ldots ,n+2}}>0$$

Despite the fact that the determinant, as follows from (12), vanishes when any of the multipliers in the middle part is equal to zero, the condition of the stability boundary must correspond to the equality to zero of one particular multiplier from this product. 

For greater clarity, let us consider a binary system, presenting $Y_{i}$ and derivatives $\frac{\partial^{2}U}{\partial Y_{i}^{2}}$ by means of specific variables:

(13) $$\left|\begin{array}{cccc} \frac{\partial T}{\partial S} & \frac{\partial T}{\partial V} & \frac{\partial T}{\partial m_{1}} & \frac{\partial T}{\partial m_{2}}\\ \frac{\partial \left(-P\right)}{\partial S} & \frac{\partial \left(-P\right)}{\partial V} & \frac{\partial \left(-P\right)}{\partial m_{1}} & \frac{\partial \left(-P\right)}{\partial m_{2}}\\ \frac{\partial \mu_{1}}{\partial S} & \frac{\partial \mu_{1}}{\partial V} & \frac{\partial \mu_{1}}{\partial m_{1}} & \frac{\partial \mu_{1}}{\partial m_{2}}\\ \frac{\partial \mu_{2}}{\partial S} & \frac{\partial \mu_{2}}{\partial V} & \frac{\partial \mu_{2}}{\partial m_{1}} & \frac{\partial \mu_{2}}{\partial m_{2}} \end{array}\right|={\left(\frac{\partial T}{\partial S}\right)}_{V,m_{1},m_{2}}\cdot {\left(\frac{\partial \left(-P\right)}{\partial V}\right)}_{T,m_{1},m_{2}}\cdot {\left(\frac{\partial \mu_{1}}{\partial m_{1}}\right)}_{T,P,m_{2}}\cdot {\left(\frac{\partial \mu_{2}}{\partial m_{2}}\right)}_{T,P,\mu_{1}}>0.$$

The multiplier ${\left(\frac{\partial \mu_{2}}{\partial m_{2}}\right)}_{T,P,\mu_{1}}$ in this relation, at first glance, is equal to zero, in accordance with the Gibbs–Duhem Equation (6). However, this is not the case, because, as before, a non-equilibrium virtual perturbation is considered, for which the inequality in (5) is fulfilled. Let us show that equality to zero of the specified derivative corresponds to the boundary of stability.

First of all, we note that the determinant in (13) can be represented as a product of derivatives in various ways. The sequence of parameter pairs is not limited to the one given in relation (13). For example, it is obvious that

(14) $$\begin{array}{c} {\left(\frac{\partial T}{\partial S}\right)}_{V,m_{1},m_{2}}\cdot {\left(\frac{\partial \left(-P\right)}{\partial V}\right)}_{T,m_{1},m_{2}}\cdot {\left(\frac{\partial \mu_{1}}{\partial m_{1}}\right)}_{T,P,m_{2}}\cdot {\left(\frac{\partial \mu_{2}}{\partial m_{2}}\right)}_{T,P,\mu_{1}}={\left(\frac{\partial \mu_{2}}{\partial m_{2}}\right)}_{S,V,m_{1}}\\ {\left(\frac{\partial \mu_{1}}{\partial m_{1}}\right)}_{S,V,\mu_{2}}\cdot {\left(\frac{\partial \left(-P\right)}{\partial V}\right)}_{S,\mu_{1},\mu_{2}}\cdot {\left(\frac{\partial T}{\partial S}\right)}_{P,\mu_{1},\mu_{2}}. \end{array}$$

Other combinations of derivatives in the transformation of the determinant in (13) are also possible. At the same time, under the condition ${\left(\frac{\partial \mu_{2}}{\partial m_{2}}\right)}_{T,P,\mu_{1}}=0$ , other similar conditions will also be valid, namely:

(15) $${\left(\frac{\partial \mu_{2}}{\partial m_{2}}\right)}_{T,P,\mu_{1}}={\left(\frac{\partial T}{\partial S}\right)}_{P,\mu_{1},\mu_{2}}={\left(\frac{\partial \left(-P\right)}{\partial V}\right)}_{T,\mu_{1},\mu_{2}}={\left(\frac{\partial \mu_{1}}{\partial m_{1}}\right)}_{T,P,\mu_{2}}=0.$$

As indicated in Gibbs’ work [1] and subsequent studies, for example [3], the relations in (15) or their consequences are, with disregard for infinitesimal higher orders, conditions of neutral equilibrium. In this case (neutral equilibrium), we can already use the results for equilibrium processes, for example [10,11,12]:

(16) $${\left(\frac{\partial X_{i}}{\partial Y_{i}}\right)}_{Y_{1},Y_{2},\cdots ,Y_{i-1},Y_{i+1},\cdots ,Y_{n+1},Y_{n+2}}\geq {\left(\frac{\partial X_{i}}{\partial Y_{i}}\right)}_{Y_{1},Y_{2},\cdots ,Y_{i-1},Y_{i+1},\cdots ,Y_{n+1},X_{n+2}}\geq \cdots \;\;\geq {\left(\frac{\partial X_{i}}{\partial Y_{i}}\right)}_{Y_{1},X_{2},\cdots ,X_{i-1},X_{i+1},\cdots ,X_{n+2}}\geq {\left(\frac{\partial X_{i}}{\partial Y_{i}}\right)}_{X_{1},X_{2},\cdots ,X_{i-1},X_{i+1},\cdots ,X_{n+2}}=0.$$

Note that in this case, taking into account the previous discussion, we have expanded the options for fixing independent parameters (in comparison with [1,2,3,10,11] and other works) by including the condition $X_{1},X_{2},\cdots ,X_{i-1},X_{i+1},\cdots ,X_{n+2}=const$ in (16). It follows from the inequality in (16) that, in particular, for derivatives in relation (13), the following conditions are satisfied at the stability boundary

(17) $${\left(\frac{\partial T}{\partial S}\right)}_{V,m_{1},m_{2}}\geq {\left(\frac{\partial T}{\partial S}\right)}_{P,\mu_{1},\mu_{2}}=0,\;\;{\left(\frac{\partial \left(-P\right)}{\partial V}\right)}_{T,m_{1},m_{2}}\geq {\left(\frac{\partial \left(-P\right)}{\partial V}\right)}_{T,\mu_{1},\mu_{2}}=0;\;\;{\left(\frac{\partial \mu_{1}}{\partial m_{1}}\right)}_{T,P,m_{2}}\geq {\left(\frac{\partial \mu_{1}}{\partial m_{1}}\right)}_{T,P,\mu_{2}}=0.$$

Thus, we have proven that all the multipliers in the ratio (16) will be greater than the last of them, ${\left(\frac{\partial \mu_{2}}{\partial m_{2}}\right)}_{T,P,\mu_{1}}$ , which determines the stability boundary. Obviously, this result can be easily generalized to the case of an arbitrary number of pairs of thermodynamic parameters. In all cases, the condition of the stability boundary is that the determinant in Equation (13) is equal to zero, or the condition

(18) $${\left(\frac{\partial^{2}U}{\partial Y_{i}^{2}}\right)}_{\;\frac{\partial U}{\partial Y_{1}},\;\;\;\frac{\partial U}{\partial Y_{2}},\;\cdots ,\;\frac{\partial U}{\partial Y_{i-1}},\;\frac{\partial U}{\partial Y_{i+1}},\cdots ,\frac{\partial U}{\partial Y_{n+2}}}={\left(\frac{\partial^{2}U}{\partial Y_{i}^{2}}\right)}_{\;X_{1},X_{2},\cdots ,X_{i-1},X_{i+1},\cdots ,X_{n+2}}=0.$$

These conditions, in general, coincide with the results of Gibbs [1] and the conclusions of subsequent works defining the stability boundary as a state of neutral equilibrium (with disregard for infinitesimal higher orders). Note that special cases where higher-order variations should be taken into account, for example, in a critical condition, require additional analysis.

## 3. Conclusions

The results are useful for analyzing the stability of the homogeneous state of a multicomponent thermodynamic system. From a methodological point of view, the novelty elements are associated with the rejection of the traditional approach to the study of stability, which requires the introduction of a “scale factor”, that is, fixing one of the extensive parameters or their complex (as a rule, the sum of the masses of the components). In addition, it has been shown that it is wrong to involve the relations, such as the Gibbs–Duhem equation, established for the equilibrium displacement in the analysis of a non-equilibrium virtual perturbation. This leads to factual and methodological errors, including those related to the general conditions of the stability boundary. A new version of the derivation of the stability boundary conditions is presented, which assumes a departure from equilibrium during a virtual perturbation of the state of the system. The final conclusions, in general, coincide with the classical results, according to which a neutral (indifferent) equilibrium is realized at the stability boundary by neglecting higher-order internal energy variations (the critical states are thereby excluded). From our point of view, the described approach provides a clearer and more accurate idea of the actual conditions of stability and its boundaries. 

## Data Availability

All necessary data are contained in the paper.

## References

[1] Gibbs J.W. (1931). The Collected Works. V. 1. Thermodynamics.

[2] Munster A. (1970). Chemical Thermodynamics.

[3] Storonkin A.V. (1967). Termodinamika Geterogennyh System (Thermodynamics of Heterogeneous Systems).

[4] Prigogine I., Defay R. (1954). Chemical Thermodynamics.

[5] Kondepudi D., Prigogine I. (2014). Modern Thermodynamics: From Heat Engines to Dissipative Structures.

[6] Tisza L. (1966). Generalized Thermodynamics.

[7] Hillert M. (2007). Phase Equilibria, Phase Diagrams and Phase Transformations: Their Thermodynamic Basis.

[8] Tester J.W., Modell M. (1997). Thermodynamics and Its Applications.

[9] Haase R. (1990). Thermodynamics of Irreversible Processes.

[10] Toikka A.M., Hong W.I. (2010). Stability of chemical and phase equilibrium: Alternative forms of equations for thermodynamic analysis. Mathematical Chemistry.

[11] Gromov D., Toikka A. (2020). On an alternative formulation of the thermodynamic stability condition. J. Math. Chem..

[12] Gromov D., Toikka A. (2020). Toward Formal Analysis of Thermodynamic Stability: Le Chatelier–Brown Principle. Entropy.

