# Correction: Toikka et al. Some Remarks on the Boundary of Thermodynamic Stability. *Entropy* 2023, *25*, 969

**DOI:** 10.3390/e26070547

**Published:** 2024-06-27

**Authors:** Alexander Toikka, Georgii Misikov, Maria Toikka

**Affiliations:** Department of Chemical Thermodynamics & Kinetics, Saint Petersburg State University, Universitetsky pr. 26, Peterhof, Saint Petersburg 198504, Russia; st062450@student.spbu.ru (G.M.); m.toikka@spbu.ru (M.T.)

The authors would like to make a tiny but important correction to the published paper [1]. Unfortunately, in the article, when quoting Gibbs’ work [2], an inaccuracy was made in writing Formula (8). The correct spelling of this formula is as follows:

(8) $$\Delta U>T\Delta S-P\Delta V+\mu_{1}\Delta m_{1}+\cdots +\mu_{n}\Delta m_{n}$$

The authors would like to apologize for any inconvenience caused by these errors. The authors state that the scientific conclusions are unaffected. This correction was approved by the Academic Editor. The original publication has also been updated.
